# Periodic Heat Stress Licenses EMSC Differentiation into Osteoblasts via YAP Signaling Pathway Activation

**DOI:** 10.1155/2022/3715471

**Published:** 2022-03-18

**Authors:** Wentao Shi, Zhe Wang, Lu Bian, Yiqing Wu, Mei HuiYa, Yanjun Zhou, Zhijian Zhang, Qing Wang, Peng Zhao, Xiaojie Lu

**Affiliations:** ^1^Jiangnan University Affiliated Hospital, Wuxi, Jiangsu Province 214122, China; ^2^School of Medicine, Jiangsu University, Zhenjiang, Jiangsu Province 212001, China; ^3^School of Medicine, Jiangnan University, Wuxi, Jiangsu Province 214122, China; ^4^Affiliated Wuxi Second Hospital, Nanjing Medical University, Wuxi, Jiangsu Province 214122, China

## Abstract

**Background:**

The repair and regeneration of large bone defects represent highly challenging tasks in bone tissue engineering. Although recent studies have shown that osteogenesis is stimulated by periodic heat stress, the thermal regulation of osteogenic differentiation in ectomesenchymal stem cells (EMSCs) is not well studied.

**Methods and Results:**

In this study, the direct effects of periodic heat stress on the differentiation of EMSCs into osteoblasts were investigated. EMSCs derived from rat nasal respiratory mucosa were seeded onto culture plates, followed by 1 h of heat stress at 41°C every 7 days during osteogenic differentiation. Based on the results of the present study, periodic heating increases alkaline phosphatase (ALP) activity, upregulates osteogenic-related proteins, and promotes EMSC mineralization. In particular, increased YAP nuclear translocation and YAP knockdown inhibited osteogenic differentiation induced by heat stress. Furthermore, the expression and activity of transglutaminase 2 (TG2) were significantly increased after YAP nuclear translocation.

**Conclusion:**

Together, these results indicate that YAP plays a key role in regulating cellular proteostasis under stressful cellular conditions by modulating the TG2 response.

## 1. Introduction

Bone defects are one of the most challenging clinical problems for orthopedists. Large bone defects are characterized by delayed bone healing or nonhealing and account for 5% and 10%, respectively, of total bone fracture cases [1]. Currently, some bone defects, particularly large bone defects, are difficult to repair, which may cause nonunion and thus affect a patient's quality of life.

Fortunately, mesenchymal stem cells (MSCs) are recruited to the fracture site after the occurrence of a fracture to facilitate repair [2, 3]. Numerous studies examining the effects of growth factors or cytokines on MSC differentiation into osteoblasts have been performed [4, 5]. Previous studies conducted in our laboratory have shown that the lamina propria of the mucosa lining the respiratory region of the nasal cavity contains ectomesenchymal stem cells (EMSCs) [6, 7]. These cells have the potential to differentiate into a variety of cell types, including osteoblasts [8, 9]. As shown in our recent study, the injection of ectomesenchymal stem cells (EMSCs) into a skull bone defect site contributes to bone defect repair [6]. However, osteoblasts differentiated from MSCs are still not as functionally mature as primary adult cells. Therefore, more effective and simplified therapeutic methods designed to improve EMSC maturation and optimize their differentiation must be identified.

MSC differentiation into mature functional osteoblasts is a complex process regulated and influenced by many factors that are broadly classified into extrinsic and intrinsic factors [10, 11]. In recent years, accumulating evidence has indicated that physical signals induced by the cellular microenvironment may control MSC behaviors through a mechanosensitive signaling network [12, 13]. Among them, temperature exerts an important effect on bone growth in vivo and in vitro. To date, no study has examined the effects of the heat stress microenvironment on the osteogenesis of EMSCs.

Yes-associated protein (YAP), which is a promoter of ligand binding, regulates signal transduction and gene transcription in various cells [14]. It also plays a vital role in regulating tissue regeneration and stem cell development [15]. According to recent studies, YAP nuclear translocation appears to be an integrator of cell differentiation and proliferation in response to changes in the microenvironment [16, 17]. Other relevant studies also confirmed that YAP was a key regulator that not only promotes osteogenesis via the BMP, Wnt, or *β*-catenin signaling pathways [18, 19] but also modulates the responses to various extracellular factors, such as cell adhesion-driven mechanical cellular stress [20]. Interestingly, a previous study reported that heat stress induces substantial YAP nuclear translocation, thereby enhancing the heat shock transcriptome and cell survival [21]. Since heat stress may modulate the nuclear translocation of YAP in many cell types, a reasonable hypothesis is that it might influence EMSC fate through YAP-mediated osteogenic differentiation. However, many key questions remain to be answered regarding heat stress therapy. For example, the signaling pathways involved are poorly understood.

Thus, we investigated the effects of periodic heat stress treatment on the osteogenesis of EMSCs to explore the potential underlying mechanism during the osteogenic differentiation process. We showed that YAP is necessary to promote EMSC osteogenic differentiation. YAP knockdown impairs EMSC osteogenic differentiation and diminishes heat-enhanced EMSC osteogenesis. Additionally, YAP controls the heat stress response by modulating the expression of transglutaminase 2 (TG2), which functions as a crosslinker of the extracellular matrix (ECM) [22], increasing both its deposition and accumulation by crosslinking ECM proteins [23, 24]. Moreover, we report that cysteamine (CYS) inhibits TG2 activity and restrains the osteogenic differentiation of EMSCs. In contrast, TG2 overexpression promotes EMSC osteogenic differentiation, thereby indicating that YAP mediates EMSC osteogenic differentiation by increasing TG2 activity, which might explain the utility of periodic heat stress as a treatment for osteogenesis.

## 2. Materials and Methods

### 2.1. Cell Culture

EMSCs were isolated from rats as described in our previous work [6]. Briefly, two rats (50-80 g) were sacrificed by administering pentobarbital sodium (200 mg/kg). Then, mucosal tissue samples were collected from the rat nasal septum. These mucous membranes were gently minced into pieces and cultured in Dulbecco's modified Eagle's medium/nutrient mixture F12 containing 10% fetal bovine serum (FBS, HyClone, NY, USA) and placed at 37°C in a humidified incubator containing 5% CO_2_. The medium was changed every 3 days, and after 7 days, EMSCs were harvested using 0.25% trypsin and subcultured until they reached 85% confluence. EMSCs (passage 3) were identified by detecting the expression of nestin (Abcam; UK), CD133 (Abcam; UK), and vimentin (Boster Biological Technology). Cell purity was determined by performing immunofluorescence staining for CD133, vimentin, and nestin. Filamentous actin (F-actin) was stained with 0.1 *μ*mol/L Acti-stain 488-conjugated phalloidin (Cytoskeleton, Inc., Denver, CO, USA), as described in a previous study.

### 2.2. Cell Viability Assay

A CCK-8 assay was conducted to assess the cytotoxicity of heat stress at different temperatures. Cells were seeded onto 96-well plates at a density of 2 × 10^3^ cells per well and then incubated for 72 h. Then, the medium was replaced with 200 *μ*L of medium containing 10 *μ*L of CCK-8. After an additional 4 h incubation, the absorbance of formazan crystals was measured with an Infinite F200 Multimode plate reader (Tecan, Crailsheim, Germany) at 490 nm. All experiments were conducted in triplicate.

### 2.3. In Vitro Neurogenic and Adipogenic Differentiation Assays

Neurogenic differentiation was induced as described in our previous study with slight modifications [9]. Briefly, EMSCs were cultured in medium containing B27 and N2 supplement, 1 mg/mL ATRA, and 20 ng/mL BDNF for 2 weeks. Then, the differentiated cells were fixed with 4% formaldehyde and analyzed for expression of the neural cell markers GAP-43 and TUBB3 using immunocytofluorescence staining. Nuclei were stained with 4′,6-diamidino-2-phenylindole (DAPI, Merck). For adipogenic differentiation, EMSCs cultured in a 24-well plate were treated with adipogenic differentiation medium (Puhe Biotechnology) according to the manufacturer's protocol. At 2 weeks, the cells were fixed with 4% formaldehyde and incubated with fresh oil red O, and lipid droplets were visualized and photographed.

### 2.4. Immunofluorescence Staining

For immunofluorescence staining, cells were fixed with 4% formaldehyde and then washed three times with PBS. Afterward, the cells were permeabilized and blocked with 0.1% Triton X-100 (Guo Yao, Shanghai, China) containing 3% bovine serum albumin (Solarbio, Beijing, China) for 30 min at room temperature. Following three washes with PBS, the cells were incubated with primary antibodies against nestin and vimentin at 4°C for 8 h. The samples were rinsed with PBS and then incubated with the appropriate Cy3- or Alexa Fluor 488-conjugated secondary antibodies at 37°C for 1 h. The nuclei were counterstained with DAPI at room temperature for 30 min, and the cells were observed under a fluorescence microscope (Zeiss, Germany).

### 2.5. Western Blot Analysis

Total protein was extracted from cells using cell lysis buffer (Boster) containing protease inhibitors (Boster, Wuhan, China). Total proteins were separated by sodium dodecyl sulfate polyacrylamide gel electrophoresis on 10% gels, and the separated proteins were transferred to polyvinylidene fluoride membranes (Millipore, NY, USA). After blocking with 3% BSA for 2 h, the membranes were incubated with primary antibodies against the following proteins: YAP (1 : 500, Boster, Wuhan, China), COL I (1 : 1000; Santa Cruz, USA), osteocalcin (OCN; 1 : 1000; Abcam, UK), RUNX-2 (1 : 1000; Abcam, UK), osteopontin (OPN; 1 : 1000; Boster), TG2 (1 : 1000; Santa Cruz, USA), HSP70 (1 : 500, Bioss, Beijing, China), and YAP (1 : 500, Boster, Wuhan, China). HRP-conjugated goat anti-rabbit IgG (1 : 5000; Boster) was applied as a secondary antibody and incubated with the membrane for 1 h at room temperature. Immunoreactive bands were detected using enhanced chemiluminescence reagents (Millipore, NY, USA). Signal intensity was measured using a Bio-Rad XRS chemiluminescence detection system (Bio-Rad, CA, USA). Actin or *β*-tubulin served as the loading control.

### 2.6. Transfection

siRNA targeting YAP was obtained from Nanjing GenScript Bioengineering Technology and Services Co., Ltd. (Nanjing, China) and transfected into EMSCs using Lipofectamine 2000 reagent (Invitrogen) according to the manufacturer's instructions. The sequences of siRNAs for the indicated target genes were as follows: CCAATAGTTCAGATCCCTT, GCATGAGCAGCTACAGCAT, and GGCAATACGGAATATCAAT. Briefly, 6 × 10^5^ cells were seeded onto 6-well plates with 2 mL of DMEM/F12 containing 10% FBS. At the same time, siRNAs or NC were mixed with the transfection reagent and incubated at room temperature for 10 min. Then, the complexes were transfected into EMSCs for 48 h. Recombinant adenovirus-mediated TG2 overexpression was performed as described previously [6]. EMSCs were infected with either Adv-Vector (as a control) or Adv-TG2 for 24 h. Cells were harvested at 24 hours postinfection.

### 2.7. Osteogenic Differentiation Protocol

For the induction of EMSC differentiation, osteogenic induction medium supplemented with 10 mM *β*-glycerophosphate, 0.1 *μ*M dexamethasone, and 50 *μ*g/mL ascorbic acid was incubated with the cells for 48 h at 37°C in the presence of 5% CO_2_. The cells were maintained by adding fresh osteogenic induction medium every 7 days. On the seventh day of osteogenic differentiation induction of EMSCs, the second round of siRNA transfection was performed using the same siRNA concentration. The expression level of YAP was also evaluated at days 7 and 14. Data are provided in the Supplementary Materials. The expression levels of bone matrix proteins and osteogenic regulatory proteins were detected using western blotting.

### 2.8. Heat Exposure

EMSCs cultured with osteogenic induction medium were exposed to periodic heat stress on days 1 and 7. Cells were differentiated under various temperature conditions to investigate the effects of temperature on the osteogenic differentiation and proliferation of EMSCs. Briefly, cells were placed in a water bath at 37, 38, 39, 40, 41, or 42°C followed by recovery to 37°C, as indicated. The medium was changed after heating, and the cells were returned to a 37°C incubator. The control samples remained in the 37°C incubator, while the medium was changed at the same time as that of the heat-shocked samples.

### 2.9. ELISA

ELISA was conducted with a TGF-*β* ELISA kit. EMSCs were cultured in a 48-well plate with 0.5 mL of osteogenic induction medium and incubated at 37°C with 5% CO_2_ and 95% humidity for 14 days. All of the supernatant was collected on days 2 and 8. ELISA was performed according to the manufacturer's protocol. Absorbance was read with a microplate reader at a wavelength of 490 nm.

### 2.10. In Vitro Differentiation Assay

Alizarin red S (ARS) staining was applied to visualize the deposition of calcium phosphate. Cells were fixed with 4% paraformaldehyde at 4°C for 8 h. The fixed cells were washed with ddH_2_O and incubated with 1 mL/well Alizarin red staining solution (0.5% (*w*/*v*) ARS (Sigma-Aldrich)) for 10 min at 37°C.

ALP staining was applied to EMSCs undergoing osteogenic differentiation to visualize the presence of preosteoblasts and osteoblasts. ALP activity in the cells was identified using an ALP staining kit (Solarbio) to stain the cells with ALP substrate solution according to the manufacturer's protocol. After staining, the samples were examined using a microscope.

### 2.11. Quantitative RT-PCR Analysis

Total RNA was extracted from EMSCs with TRIzol (Invitrogen) according to the manufacturer's instructions. First-strand cDNA was prepared by reverse transcription with Superscript II reverse transcription (Invitrogen) and oligo(dT) primers and stored at -20°C. Quantitative PCR was performed with a CFX96 Touch Real-Time PCR system (Bio-Rad) using 0.5 *μ*L of cDNA. Ubiquitously expressed *β*-actin was used as an endogenous control. As an internal control, the mRNA levels of GAPDH were quantified in parallel with the mRNA levels of the target genes. Primer sets are listed in the supplementary information (Table S1).

### 2.12. Statistical Analysis

Statistical analyses were performed using SPSS 22.0 software (IBM, USA). All data are presented as the mean ± standard deviation (mean ± SD). *P* < 0.05 was considered significant. The significance of differences between groups was assessed using two-way analysis of variance (ANOVA) with Tukey's post hoc analysis.

## 3. Results and Discussion

### 3.1. Immunophenotypes of Purified EMSCs Grown on a Plastic Surface

**Figure 1:**
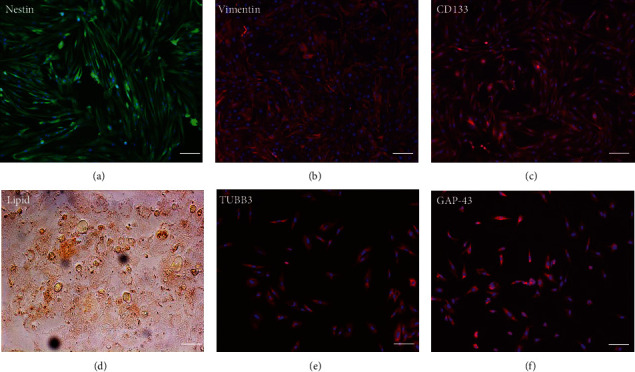
Isolation and characterization of EMSCs. (a–c) Immunostaining of tendon-derived cells using antibodies against nestin, vimentin, and CD133. Multilineage differentiation potential: (d) oil red O staining (adipogenesis) and (e, f) TUBB3 and GAP-43 immunostaining for EMSC neural differentiation. Bar = 25 *μ*m.

Primary cultures of rat EMSCs have been widely used in studies of various diseases. Immunostaining was performed to assess the expression of three markers, nestin, vimentin, and CD133, to determine the immunophenotypic profiles of the purified cells (Figure 1). Nestin is a class VI intermediate filament protein that is a marker of neural or neural crest stem cells and is a potential mesenchymal stem cell marker. Meanwhile, the cell surface antigens CD133 and vimentin are considered markers of stem cells, including EMSCs. The purification procedure ensured that a high percentage of CD133-, vimentin-, and nestin-positive cells were present in the cultured population, indicating that passage 3 EMSCs were of high purity.

### 3.2. Multilineage Differentiation of EMSCs

One of the defining characteristics of stem cells is their ability to differentiate into cells of other lineages [25]. Therefore, we determined the ability of nasal mucosa-derived EMSCs to differentiate into neuron-like cells and adipocytes. The ability of EMSCs to differentiate into neuron-like cells was determined by staining for GAP-43 and TUBB3 in cells following neural differentiation induction (Figure 1). The results of immunofluorescence staining showed that the EMSCs were strongly positive for GAP-43 and TUBB3, which are typical markers of neural cells [26, 27]. After 14 days of in vitro differentiation, lipid vacuoles were observed in cultures cultured in adipogenic differentiation medium and stained positively with oil red O (Figure 1). These results implied that the cells isolated from nasal mucosa tissues had similar biological characteristics to MSCs and possessed the potential for multilineage differentiation. These results were consistent with our previous studies [9]. Thus, the cells that we isolated and cultured from nasal mucosa tissue were defined as EMSCs.

### 3.3. In Vitro Assessments of Cell Viability and Proliferation

**Figure 2:**
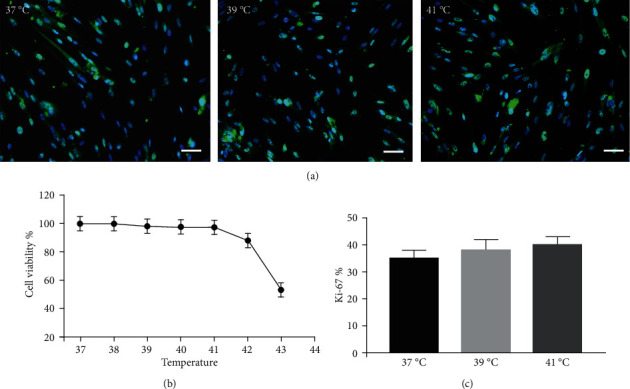
Effect of heat stress at different temperatures on EMSC proliferation. After 72 hours of treatment with heat stress, (a) Ki-67 expression was determined using immunofluorescence staining, and 200 cells cultured under each condition from at least 6 independent fields were counted. Ki-67-positive cells are presented as a percentage of the total population. (b) Cell proliferation was measured using the CCK-8 assay. (c) Graphs showing the percentage of Ki-67-positive cells, bar = 25 *μ*m.

**Figure 3:**
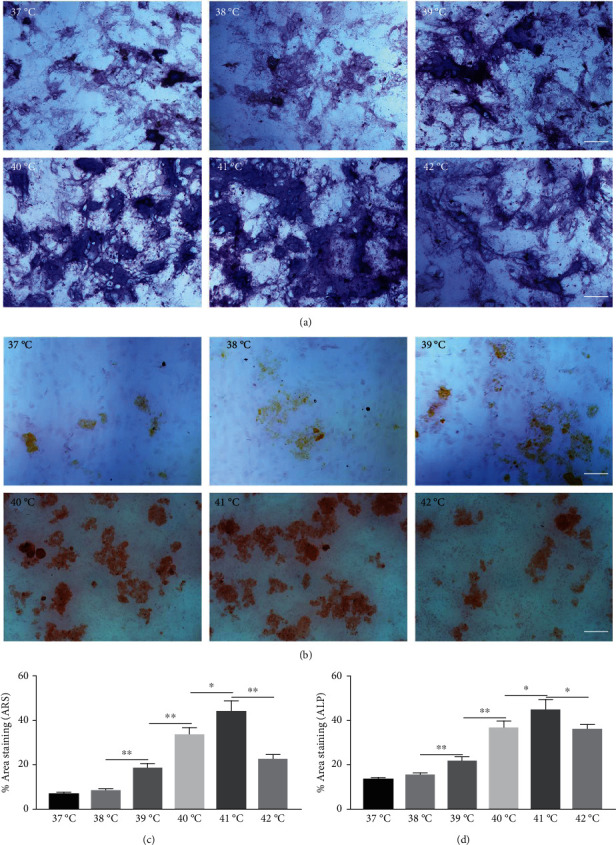
Effects of different heat stresses on the osteogenic differentiation of EMSCs cultured in osteogenic medium after 7 days using ALP and Alizarin red S staining. (a, b) Osteogenic differentiation was determined by performing ARS and ALP staining to indicate mineralization and differentiation, respectively. Measured areas of mineralized nodules from (c) ARS staining and (d) ALP activity. EMSCs exposed to heat stress at 41°C had larger areas of mineralized nodules and ALP activity, indicating greater osteogenic differentiation. Data are presented as the means ± SD of at least three independent experiments. ^∗^*P* < 0.05 and ^∗∗^*P* < 0.01.

The effect of different heat stresses on the viability of EMSCs was measured by performing the MTT assay and a quantitative analysis of Ki-67 staining. EMSCs were cultured for 3 days under different temperature conditions (normal medium), and we found that heat stress (<41°C) did not induce significant cytotoxicity, while a high temperature (>41°C) was not suitable for EMSCs (Figure 2). Moreover, we performed Ki-67 staining to confirm the proliferation of EMSCs under heat stress at different temperatures. Ki-67 staining normalized to the DAPI signal was not significantly different between EMSCs from the heat stress-treatment and control groups (Figure 2). Osteogenic differentiation assays were performed using ESMCs to determine the most appropriate temperature of heat stress for EMSC osteogenic differentiation in vitro. EMSCs treated with different temperatures were incubated with osteogenic induction medium for 14 days. Significantly higher ALP activity was detected in the groups incubated at 41°C than in other groups (Figure 3(a)), and the statistical comparison is depicted in Figure 3(c). Obvious calcium nodules were also observed in the groups incubated at 41°C (Figure 3(b)), and the statistical comparison is shown in Figure 3(d). Therefore, we chose 41°C as the most appropriate temperature to induce heat stress for in vitro studies.

### 3.4. Periodic Heat Stress Promotes EMSC Osteogenic Differentiation

**Figure 4:**
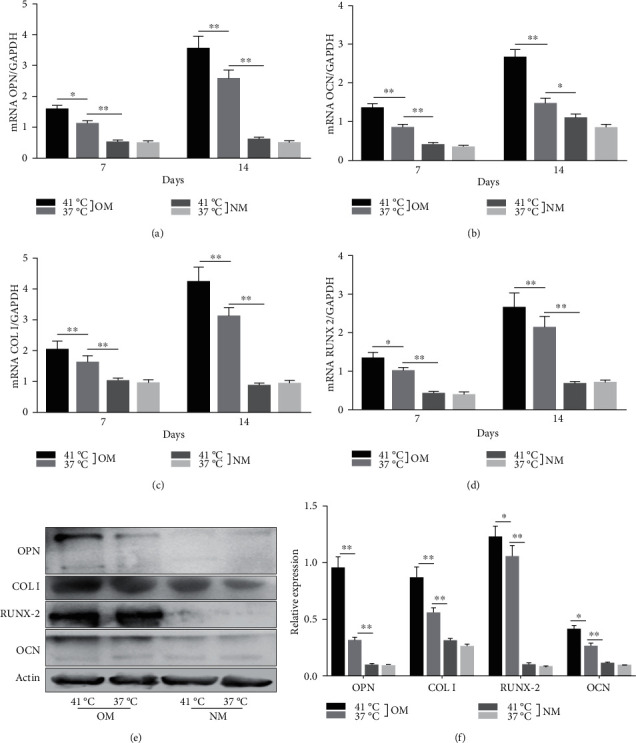
Osteogenesis of EMSCs. The expression of osteogenic genes, including OPN, OCN, COL I, and RUNX2, was increased substantially in the heat stress-treatment group compared with the control groups after osteogenic differentiation for 7 days and 14 days. (e) Increased levels of osteogenesis-associated proteins in heat stress-treated EMSCs. (f) Changes in the expression of bone-specific proteins on day 14 of differentiation, as detected by western blotting. OM = osteogenic-inducing media; NM = normal media. ^∗^*P* < 0.05 and ^∗∗^*P* < 0.01.

**Figure 5:**
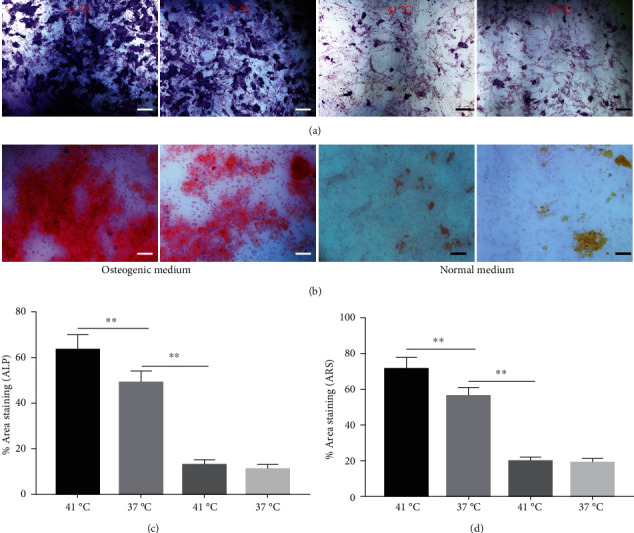
Heat stress promotes the osteogenesis of EMSCs. (a, b) ALP and ARS staining of EMSC cultures after 14 days of differentiation induction in osteogenic medium or normal medium. (c, d) Densitometry analysis of the relative ALP activity and mineralized nodules in the images. ^∗^*P* < 0.05 and ^∗∗^*P* < 0.01.

Heat shock is a common physiological and pathological stress that has been extensively studied. Several in vitro studies analyzed the possible roles of heat stress in enhancing osteogenesis [28–31], indicating that heat stress significantly enhanced the osteogenic differentiation of stem cells. We first analyzed the levels of osteogenesis-associated proteins and genes during the osteogenic differentiation of EMSCs to begin investigating the role of periodic heat stress in the regulation of EMSC osteogenic differentiation. Levels of bone-related proteins (Figure 4), including OCN, OPN, RUNX-2, and COL I, were markedly increased during osteogenic differentiation under heat stress conditions. OPN, COL I, and OCN are the most specific markers of osteogenic cells [31, 32]. OCN, COL I, and OPN were expressed at significantly higher levels in differentiated EMSCs upon exposure to heat stress-conditioned media than in cells exposed to normal media at day 14 postinduction. Additionally, the expression of the RUNX-2 protein may indicate enhanced mineralization in EMSCs (Figure 4). This result suggested that conditioned media stimulated OPN, COL I, RUNX-2, and OCN expression and the subsequent osteogenesis of EMSCs. These results were further confirmed by RT-PCR of EMSCs undergoing osteogenic differentiation (Figure 4). Next, we intended to evaluate ALP activity, as an early marker of osteogenesis, to reflect the degree of osteogenic differentiation on day 14. Compared with the control groups (Figure 5(a)), higher ALP activity was observed in the heat stress-treatment group (Figure 5(c)). Calcium deposits were also examined by ARS, and stained areas were quantified by measuring the absorbance at 560 nm. As shown in Figure 5(b), more calcium deposits appeared in the heat stress-treatment group than in the control groups on day 14 (Figure 5(d)). Altogether, the expression of osteogenic markers revealed that heat stress has the potential to promote the osteogenic differentiation of EMSCs.

### 3.5. YAP Controls the Cell Fate of EMSCs

**Figure 6:**
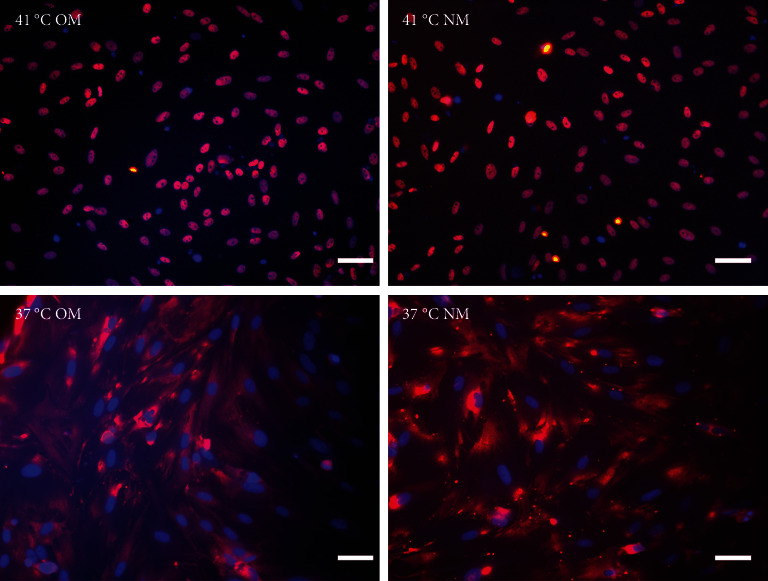
Heat shock activates YAP and induces YAP nuclear localization. EMSCs were subjected to heat shock at 41°C or 37°C for 1 min, fixed, immunofluorescence stained with anti-YAP antibodies, and examined under a microscope. Representative pictures from three independent samples are shown. Bars = 25 *μ*m.

How do heat stress conditions control the cell fate decision of EMSCs? Some studies have indicated that heat shock always activates YAP, regardless of the cell type or culture conditions, suggesting that YAP has important physiological roles in the heat stress response [33]. Moreover, a study indicated that knockout of YAP in young adult mice stabilizes nuclear *β*-catenin to drive osteoblastic differentiation of MSCs [34]. Thus, we used two experimental approaches to further test the function of YAP in EMSC differentiation under heat stress conditions. First, the effect of heat stress on promoting the nuclear translocation of YAP was confirmed by immunofluorescence staining. Our results suggested that heat stress treatment significantly promoted the nuclear translocation of YAP (Figure 6(a)). Some cells showed a high level of nuclear translocation, while cells in the control group displayed very low levels of nuclear YAP. In addition, the level of YAP nuclear translocation was significantly higher in EMSCs exposed to heat treatment than in the control groups (Figure 6(b)). Notably, osteogenic medium had no effect on the nuclear translocation of YAP. In a previous study, it was reported that enhanced nuclear translocation of YAP ultimately resulted in an increase in osteogenesis [35]. Collectively, these results indicated that heat stress enhances the osteogenic differentiation of EMSCs, at least partially by activation of the YAP signaling pathway.

**Figure 7:**
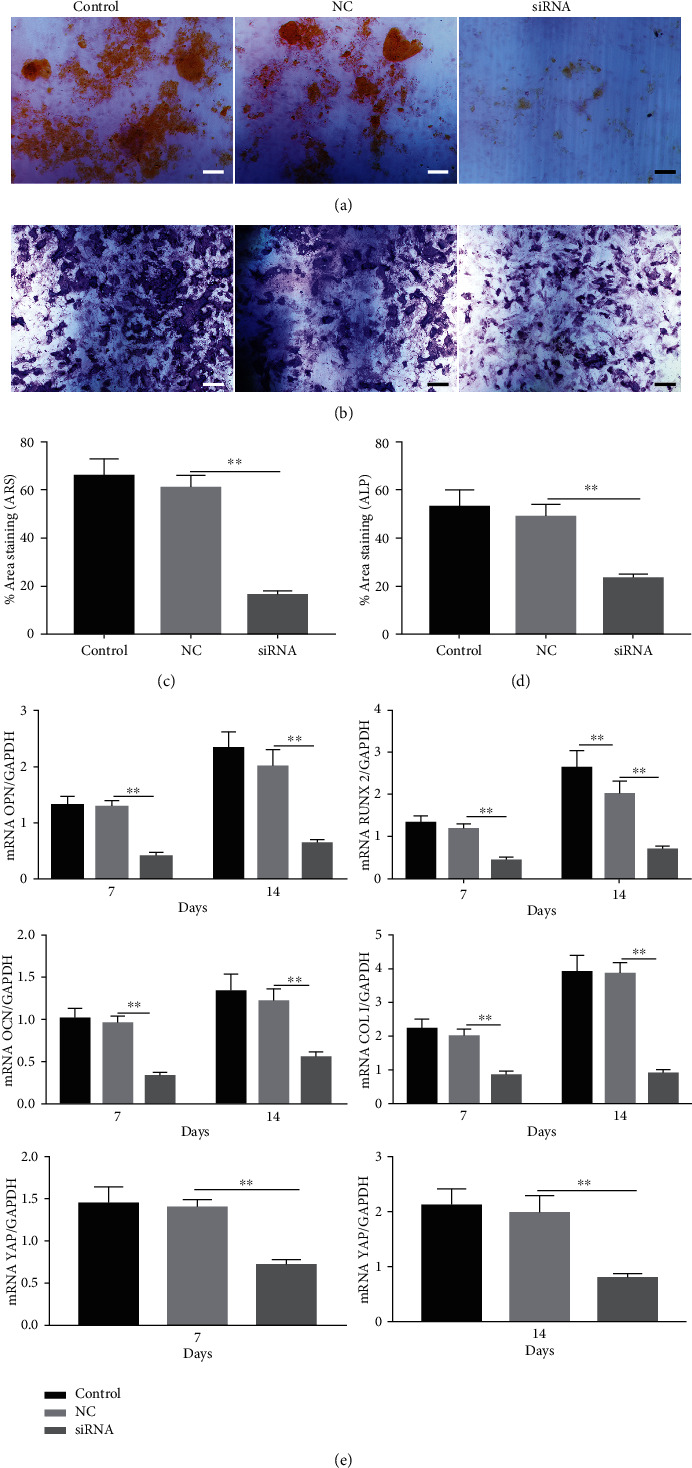
YAP silencing and its effects on osteogenic differentiation. EMSCs transfected with YAP siRNA and control siRNA were cultured in osteogenic differentiation medium for 14 days. Representative images of (a) ARS and (b) ALP activity showing the mineralized bone matrix on day 14. (c, d) Quantification of the area of calcium mineral nodules and ALP activity. (e) Expression of osteogenic-related genes in transfected EMSCs assayed using real-time qPCR on days 7 and 14 after differentiation. ^∗^*P* < 0.05 and ^∗∗^*P* < 0.01. Bars = 25 *μ*m.

Next, we cultured YAP knockdown EMSCs and control EMSCs in osteogenic differentiation induction media under heat stress conditions. YAP expression in EMSCs transfected with YAP siRNA was detected using western blotting (Figure S1A), and the protein expression level of YAP was also evaluated at days 7 and 14 (Figure S1B). Interestingly, control EMSCs easily differentiated into osteocytes after culturing in osteogenic differentiation medium for 14 days with heat stress treatment, as evidenced by strong ARS (Figure 7(a)) and ALP (Figure 7(b)) staining. However, we found that YAP silencing exerted a strong negative regulatory effect on the osteogenic differentiation of EMSCs under the same conditions. Consistent with ARS and ALP staining (Figures 7(c) and 7(d)), YAP silencing obviously reduced the levels of the OCN, OPN, RUNX-2, YAP, and COL I transcripts (Figure 7(e)). Moreover, physical stimulation, including hierarchical structure and fluid shear stresses, could enhance osteogenesis through YAP activation. These studies confirmed that YAP plays an important role in osteogenic differentiation and bone formation [36, 37]. In this study, our results revealed a positive role of YAP in EMSC osteogenic differentiation in vitro. Furthermore, this effect was enhanced by heat stress treatment.

### 3.6. Heat Stress Modifies EMSC Morphology to Regulate Cellular YAP Localization

How does heat stress regulate YAP? YAP distribution is regulated by cell morphology [38, 39]. Therefore, we examined the correlation between cell morphology and YAP distribution in EMSCs cultured under different conditions. Cell morphology was determined by staining for cytoskeletal proteins, including F-actin. Thus, we next examined the F-actin distribution in EMSCs with Alexa Fluor 488-conjugated phalloidin. As shown in Figure S2, after exposing EMSCs to heat stress for 1 h, the F-actin fibers were thick and abundant, regardless of the culture medium. However, in cells cultured at 37°C, F-actin fibers were thinner and sparser than in the abovementioned EMSCs. Studies have shown that F-actin reduces YAP phosphorylation and promotes YAP nuclear translocation downstream of changes in the morphology of mouse embryonic fibroblast NIH3T3 cells [34]. Moreover, heat stress induces LATS1 dephosphorylation and degradation and therefore reduces YAP phosphorylation [39, 40]. The ensuing decrease in YAP phosphorylation leads to an increase in the nuclear translocation of YAP in multiple cell lines [41]. However, these findings require further investigation. Collectively, these results confirm the critical role of heat stress in YAP regulation in EMSCs.

### 3.7. YAP Promotes EMSC Osteogenic Differentiation by Upregulating TG2

How does YAP nuclear translocation promote EMSC osteogenic differentiation? Recent studies have noted that TG2 may be a direct target gene of YAP [42, 43]. TG2 is a member of a group of protein-crosslinking enzymes that create large protein polymer substrate proteins by forming covalent bonds [44], resulting in modulation of cell adhesion [45], cell proliferation, and differentiation [46]. TG2 has been identified as an important extracellular crosslinking enzyme involved in ECM turnover and bone differentiation [47]. As shown in our previous study, TG2 overexpression enhances the osteogenic differentiation of EMSCs, partially by crosslinking ECM and BMP-2 [6]. Using various cell lines, Fisher and Liu showed that TG2 is directly regulated by YAP [42, 43]—by upregulating TG2 expression, YAP probably forms a positive feedback loop. However, these cell lines did not include EMSCs; therefore, we next explored whether enhanced YAP nuclear translocation induces TG2 expression and subsequently promotes EMSC osteogenic differentiation. We silenced YAP in EMSCs to further test this hypothesis, and YAP-silenced cells expressed much lower levels of TG2 and YAP than control EMSCs (Figure S3). Importantly, our data indicate that TG2 levels are significantly increased upon YAP nuclear translocation.

The forced increase in TG2 expression in EMSCs mediated by infection with an adenovirus TG2 expression vector dramatically enhanced the ability of EMSCs to differentiate into osteoblasts (Figure S4). In contrast, inhibition of TG2 by CYS in EMSCs decreased EMSC osteogenic differentiation. Based on the results from these in vitro experiments, upregulated TG2 accounted for the enhanced osteogenic differentiation potential of EMSCs. According to recent studies, TG2 interacts with *α*6/*β*4 integrins to increase FAK/Src signaling and suppress LATS signaling, subsequently leading to increased nuclear accumulation of YAP [42, 43] and probably also forming a positive feedback loop in carcinoma cancer stem cells. Overall, YAP controls EMSC osteogenic differentiation primarily by upregulation of TG2.

### 3.8. Heat Shock Increases HSP Synthesis

**Figure 8:**
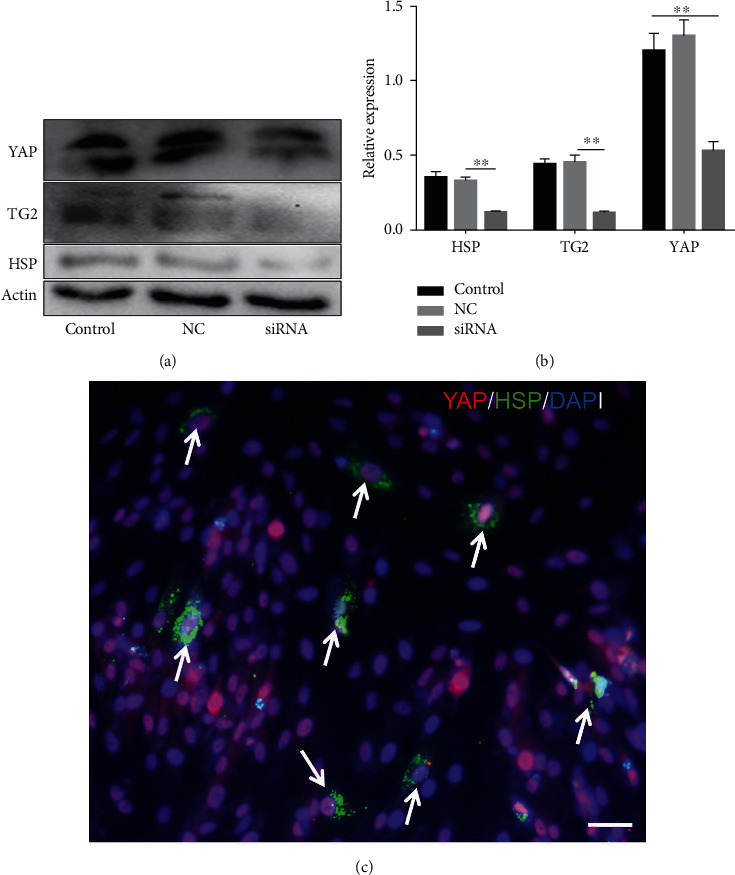
YAP induces TG2 and HSP activation. Heat shock induces YAP nuclear translocation and expression. YAP, HSP, and TG2 levels were detected by western blotting (a), and representative pictures from three independent samples are shown (b). (c) HSP colocalizes with YAP in the nucleus.

Various transcription factors and signaling molecules, including TGF-*β*, Hedgehog, and the heat shock protein (HSP) family, participate in bone development [48]. Among these proteins, HSPs are believed to play an important role in bone formation [49]. In addition, appropriate heat stress promotes the osteogenic differentiation of MSCs. ELISAs were performed on media collected from induced cells under heat stress treatment to investigate whether heat stress altered the synthesis of HSPs during the progression of EMSC osteogenic differentiation. Compared with the controls, treatment with heat stress resulted in a significant 2.8-fold increase in HSP levels, which were reduced to control levels by YAP siRNA treatment (Figure S5). Moreover, western blot results indicated that heat stress caused a significant increase in the synthesis of HSP, as observed in whole-cell lysates, while siRNA-treated cells showed the opposite results (Figure 8(a)). In addition, our results also showed colocalization of HSP and YAP in the nucleus after heat stress (Figure 8(b)). Although these results indicated that HSP is activated by heat shock during EMSC osteogenic differentiation, the signaling pathways involved are also poorly understood. As mentioned above, our results and previous studies showed that TG2 is a direct target gene of YAP. After heat stress treatment, the expression of TG2 was upregulated. Interestingly, TG2 plays a key role in regulating the cellular status by modulation of the heat-stress response and is required for the optimal activation of heat stress factors. Overall, additional mechanisms contribute to heat stress-induced regulation of the Hippo-YAP pathway for EMSC osteogenic differentiation; thus, future studies are warranted.

## 4. Conclusions

In summary, our findings indicate that the expression and function of YAP play important roles in the adaptation of EMSCs to heat stress and suggest that strategies targeting the Hippo pathway may enhance the clinical effectiveness of hyperthermia therapy for bone defects. In addition, an earlier manuscript of this paper was posted as a preprint on “Research Square” [50].

## Data Availability

The authors confirm that the data supporting the findings of this study are available within the article [and/or] its supplementary materials.

## Abbreviations

EMSCs: Ectomesenchymal stem cells
ALP: Alkaline phosphatase
TG2: Transglutaminase 2
YAP: Yes-associated protein
CYS: Cysteamine
OCN: Osteocalcin
OPN: Osteopontin
COL I: Collagen I
HSP: Heat shock protein
TGF-*β*: Transforming growth factor-*β*.

## Supplementary Materials

Supplementary Materials Figure S1: western blot showing the effectiveness of YAP knockdown in EMSCs (A). Expression level of YAP protein and YAP mRNA was suppressed in siRNA-transfected EMSCs in each time point (day 7, day 14). Figure S2: the expression of YAP and TG2 was analyzed using western blotting. Figure S3: merged images showing YAP (red), TRITC (phalloidin), and UV (DAPI) fluorescence. Figure S4: overexpression of TG2 promoted EMSC osteogenesis. On day 14 postinduction, the calcified nodules and ALP activity were visualized using ARS and ALP staining, respectively, and the results showed that overexpression of TG2 promoted EMSC osteogenesis. Figure S5: cell supernatants were monitored for HSP secretion using ELISA. Table S1: primer sequence for qRT-PCR.
