# Supplementary material for: Periodic Heat Stress Licenses EMSC Differentiation into Osteoblasts via YAP Signaling Pathway Activation
Source: Stem Cells Int. 2022 Mar 18;2022:3715471. doi: 10.1155/2022/3715471 (PMC8960005; doi:10.1155/2022/3715471)
Supplement: Supplementary Materials — Figure S1: western blot showing the effectiveness of YAP knockdown in EMSCs (A). Expression level of YAP protein and YAP mRNA was suppressed in siRNA-transfected EMSCs in each time point (day 7, day 14). Figure S2: the expression of YAP and TG2 was analyzed using western blotting. Figure S3: merged images showing YAP (red), TRITC (phalloidin), and UV (DAPI) fluorescence. Figure S4: overexpression of TG2 promoted EMSC osteogenesis. On day 14 postinduction, the calcified nodules and ALP activity were visualized using ARS and ALP staining, respectively, and the results showed that overexpression of TG2 promoted EMSC osteogenesis. Figure S5: cell supernatants were monitored for HSP secretion using ELISA. Table S1: primer sequence for qRT-PCR. [file 3715471.f1.pdf]

Supplementary information

Periodic heat stress licenses EMSC differentiation into osteoblasts via YAP signaling pathway activation

Wentao Shi[1,#], Zhe Wang[3,#], Lu Bian[1,#], Yiqing Wu[1], HuiYa, Mei[1], Yanjun Zhou[2], Zhijian Zhang[3], Qing Wang[4], Peng Zhao[2,$], Xiaojie Lu[1,2,$]

[1] Jiangnan University Affiliated Hospital, Wuxi, Jiangsu Province, 214122, P. R. China

[2] School of Medicine, Jiangnan University, Wuxi, Jiangsu Province, 214122, P. R. China

[3] School of Medicine, Jiangsu University, Zhenjiang, Jiangsu Province, 212001, P. R. China

[4]Affiliated Wuxi Second Hospital, Nanjing Medical University, Wuxi, Jiangsu Province, 214122, P. R. China

[$] Correspondence to Xiaojie Lu. E-mail: luxiaojiewuxi@163.com or xiaojielu@jiangnan.edu.cn

[#] Wentao Shi, Zhe Wang and Lu Bian contributed equally to this work.

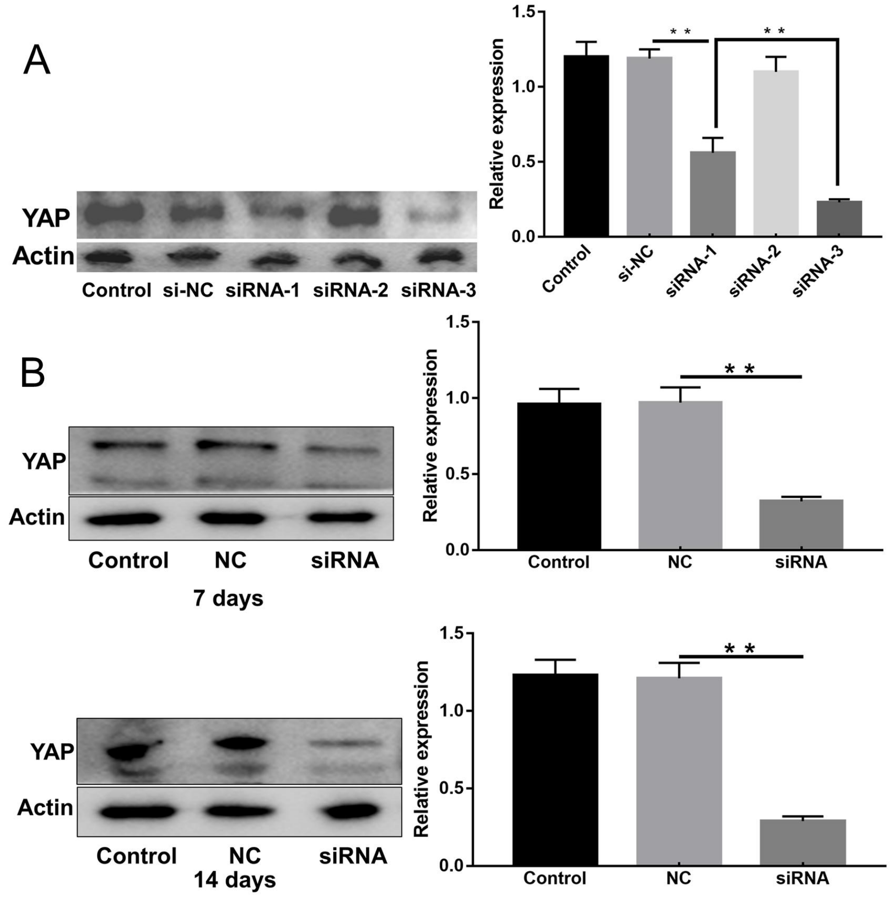

Fig. S1 (A) Western blot showing the effectiveness of YAP knockdown in EMSCs. (B) Expression

level of YAP was suppressed in siRNA-transfected EMSCs in each time point (Day 7, Day 14).

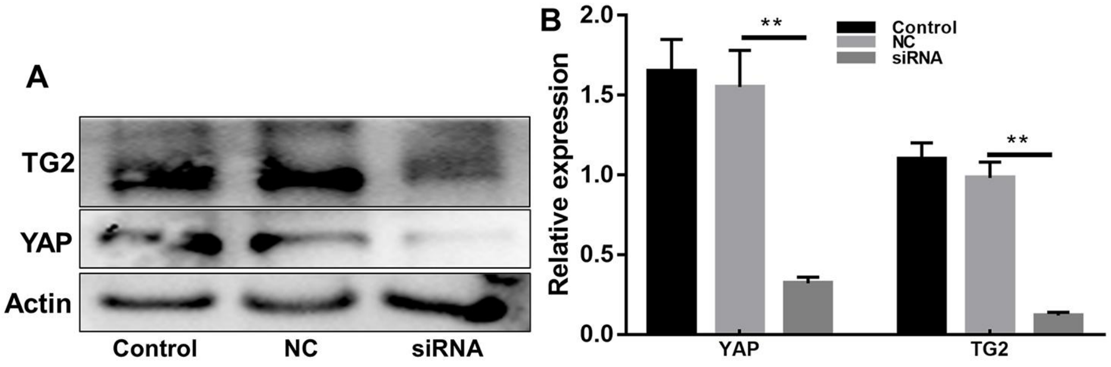

Fig. S2 The expression of YAP and TG2 was analyzed using Western blotting.

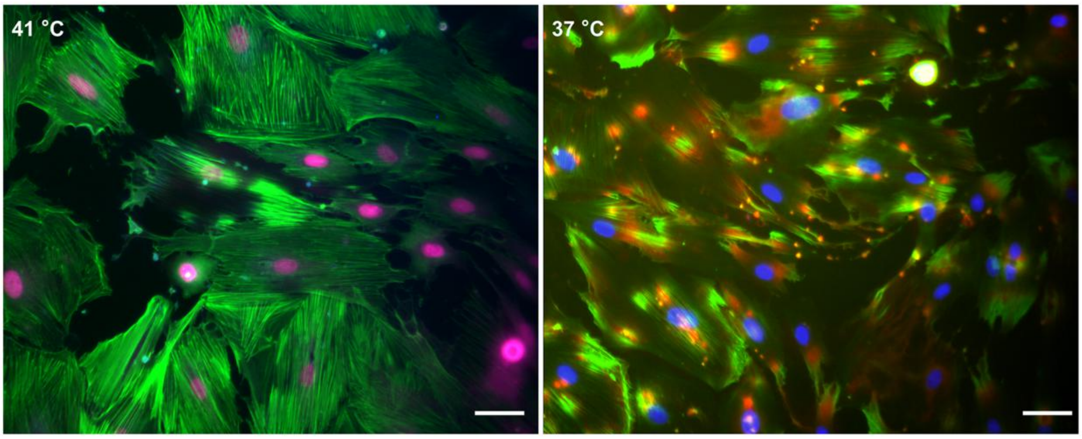

Fig. S 3 Merged images showing YAP (red), TRITC (phalloidin) and UV (DAPI) fluorescence.

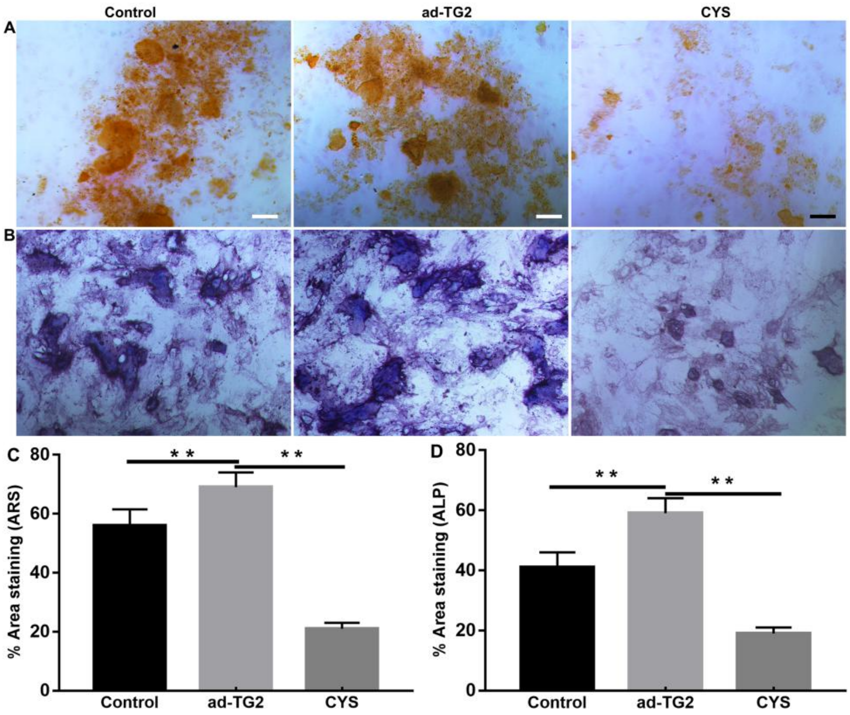

Fig. S4 Overexpression of TG2 promoted EMSC osteogenesis. On day 14 postinduction, the calcified nodules and ALP activity were visualized using ARS and ALP staining, respectively, and the results showed that overexpression of TG2 promoted EMSC osteogenesis.

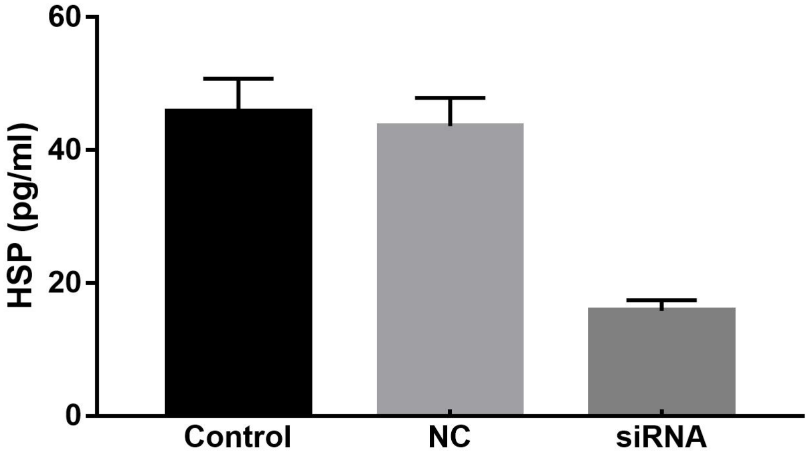

Fig. S 5 Cell supernatants were monitored for HSP secretion using ELISA.

Table S1. Primer sequence for qRT-PCR.

Gene	Forward primer sequence (5’–3’)	Reverse primer sequence (5’–3’)

OPN	TGTGAAACTCGTGGCTCTGA	GAACCAAGCGTGGAAACACA
RUNX2	TCATGGCCGGGAATGATGAG	CGCTCCGGCCTACAAATCTC
OCN	ACAGTGACCTGAGTGAGGGT	GGGACTCCTGGCTGTTCATC
COL 1	CCTGGTGCTGATGGACAACC	TTTTAGCCCAAGGGTGAAGGG
GAPDH	ACATTGGGGGTAGGAACACG	GCCATCACTGCCACTCAGAA
YAP	ACCATAAGAACAAGACCACATCC	TTCAATCGCAGCCTCTCCTT
